# Per- and Polyfluoroalkyl Substance Exposure Combined with High-Fat Diet Supports Prostate Cancer Progression

**DOI:** 10.3390/nu13113902

**Published:** 2021-10-30

**Authors:** Ozan Berk Imir, Alanna Zoe Kaminsky, Qian-Ying Zuo, Yu-Jeh Liu, Ratnakar Singh, Michael J. Spinella, Joseph Irudayaraj, Wen-Yang Hu, Gail S. Prins, Zeynep Madak Erdogan

**Affiliations:** 1 Division of Nutritional Sciences, University of Illinois, Urbana-Champaign, Urbana, IL 61801, USA; berk.imir@gmail.com; 2 Department of Food Science and Human Nutrition, University of Illinois, Urbana-Champaign, Urbana, IL 61801, USA; azk2@illinois.edu (A.Z.K.); qzuo2@illinois.edu (Q.-Y.Z.); jayliu1117@gmail.com (Y.-J.L.); 3 Comparative Biosciences, University of Illinois, Urbana-Champaign, Urbana, IL 61801, USA; rsingh02@illinois.edu (R.S.); spinella@illinois.edu (M.J.S.); 4 Cancer Center at Illinois, University of Illinois, Urbana-Champaign, Urbana, IL 61801, USA; jirudaya@illinois.edu; 5 Institute of Genomic Biology, University of Illinois, Urbana-Champaign, Urbana, IL 61801, USA; 6 Beckman Institute of Technology, University of Illinois, Urbana-Champaign, Urbana, IL 61801, USA; 7 Department of Bioengineering, University of Illinois, Urbana-Champaign, Urbana, IL 61801, USA; 8 Departments of Urology, Pathology and Physiology, College of Medicine, University of Illinois Chicago, Chicago, IL 60612, USA; wyhu@uic.edu (W.-Y.H.); gprins@uic.edu (G.S.P.); 9 Chicago Center for Health and Environment, University of Illinois at Chicago, Chicago, IL 60612, USA

**Keywords:** prostate cancer, high-fat diet, PFAS, metabolism

## Abstract

Per- and polyfluoroalkyl substances (PFAS) are synthetic chemicals utilized in various industrial settings and include products such as flame retardants, artificial film-forming foams, cosmetics, and non-stick cookware, among others. Epidemiological studies suggest a link between increased blood PFAS levels and prostate cancer incidence, but the mechanism through which PFAS impact cancer development is unclear. To investigate the link between PFAS and prostate cancer, we evaluated the impact of metabolic alterations resulting from a high-fat diet combined with PFAS exposure on prostate tumor progression. We evaluated in vivo prostate cancer xenograft models exposed to perfluorooctane sulfonate (PFOS), a type of PFAS compound, and different diets to study the effects of PFAS on prostate cancer progression and metabolic activity. Metabolomics and transcriptomics were used to understand the metabolic landscape shifts upon PFAS exposure. We evaluated metabolic changes in benign or tumor cells that lead to epigenomic reprogramming and altered signaling, which ultimately increase tumorigenic risk and tumor aggressiveness. Our studies are the first in the field to provide new and clinically relevant insights regarding novel metabolic and epigenetic states as well as to support the future development of effective preventative and therapeutic strategies for PFAS-induced prostate cancers. Our findings enhance understanding of how PFAS synergize with high-fat diets to contribute to prostate cancer development and establish an important basis to mitigate PFAS exposure.

## 1. Introduction

Per- and polyfluoroalkyl substances (PFAS) are fluorocarbons with a carbon backbone flanked with fluorine atoms and capped with a carboxyl group. PFAS adhere to metal, plastic, or other charged surfaces via an electronegative carboxylic acid group and polymerize with other PFAS compounds via fluorine atoms on their long carbon chains, forming a surface coating [1,2,3,4]. PFAS are used in many industries as a stain-repellant coating on food packaging, on cooking equipment such as non-stick pans, and to coat pipes to make them leak-proof [2]. 

Some of the most common ways that humans are exposed to PFAS are: soil, water, and air contamination; direct contact with coated surfaces; and consumption of food with contaminant exposure [5,6,7]. PFAS do not break down easily, hence these compounds are very persistent in both the environment and the human body. Due to their low manufacturing cost and wide range of uses, PFAS come in many forms. Perfluorooctane sulfonate (PFOS), perfluorobutane sulfonic acid (PFBS), and perfluorooctanoic acid (PFOA) are among the most commonly used in industry and the most environmentally persistent of these pollutants. 

Exposure to PFAS has adverse health effects, although data from animal models and epidemiology studies are not entirely consistent and conclusive. Nonetheless, many environmental and public health institutions, including the Environmental Protection Agency and the National Institutes of Health, have issued advisories regarding the possible health effects of PFAS exposure [8,9]. In response, many industries have begun to market PFAS alternatives to replace these flagged chemicals. Other frequently used PFAS, such as perfluorononanoic acid (PFNA), perfluorohexane sulfonic acid (PFHxS), and perfluorodecanoic acid, were developed to circumvent the banned chemicals but have also been determined to persist as environmental contaminants in drinking water and surface soil [5]. 

PFAS exposure causes numerous cellular and systemic metabolic alterations. Mice exposed to PFAS have altered liver metabolism with a significant shift in amino acid and citric acid cycle-dependent energy metabolism [10,11]. Specifically, PFOS exposure significantly upregulates peroxisomal *β*-oxidation-controlling enzymes [12]. Exposure to high PFOS concentrations results in higher peroxisome, endoplasmic reticulum, mitochondria, and membrane protein concentrations [13]. Further, PFAS exposure dysregulates multiple lipid metabolism pathways (glycosphingolipid metabolism, fatty acid metabolism, de novo lipogenesis, and linoleic acid metabolism) as well as amino acid metabolic pathways (aspartate and asparagine, tyrosine, and arginine and proline metabolism) [14,15]. Therefore, PFAS can impact lipid metabolism and alter cellular energetics, which can have a detrimental impact on health outcomes. In addition to cellular shifts observed in vitro and in vivo, PFAS trigger systemic changes in metabolism as well. Genome-wide association studies in PFAS-exposed individuals have shown a positive correlation between PFAS and metabolites such as fatty acids and glycerophospholipids [16]. Further, children with low to moderate serum PFOA, PFOS, PFNA, and PFHxS concentrations exhibit altered arginine, proline, aspartate, asparagine, butanoate, glycine, serine, alanine, and threonine metabolism [17]. 

Prostate cancer is the most common male cancer in the United States, with 248,530 new cases and 34,130 deaths predicted in 2021, and a prevalence of one in every eight men with a lifetime risk of prostate cancer diagnosis [18]. Epidemiology studies suggest an increase in prostate cancer incidence and/or mortality with increasing years of chronic occupational PFAS exposure or living in regional PFAS hotspots [19,20,21,22,23,24,25], particularly in men with familial prostate cancer risk, suggesting a gene-environment interaction [25]. However, whether PFAS exposures initiate carcinogenesis or promote progression of latent or later stage prostate cancer is unknown. 

In addition, compelling evidence from human prostate cell lines and transgenic murine prostate cancer models indicates that a high-fat diet (HFD) contributes to prostate cancer progression by shifting the prostate metabolome to a pro-cancerous state [26,27]. However, the cellular targets and potential mechanisms of PFAS contribution to prostate cancer are unknown. 

To explore the mechanism of PFAS action in the prostate, we performed the first mechanistic studies that used a combination of human prostate cells and relevant human prostate cancer models, spatial and molecular data, and sophisticated analytical tools to identify key mechanistic insights into prostate carcinogenesis as a function of PFAS and HFD exposures. 

## 2. Materials and Methods

### 2.1. Cell Culture and Viability Assays

Congenic RWPE-1 (non-tumorigenic) and RWPE-kRAS (tumorigenic, derived from RWPE-1 with K-ras oncogene transfection) cells were purchased from ATCC (RWPE-1, #CRL-11609; RWPE-kRAS, #CRL-11610) and maintained in Gibco Keratinocyte SFM 1X growth media with glutamine (Gibco 17005042, Fisher Scientific, Waltham, MA, USA). The day before treatments, cells were seeded at a density of 5000 cells/well in a 96-well plate. The next day, cells were treated with varying concentrations (10^−5^ M, 10^−6^ M, 10^−7^ M, 10^−8^ M, 10^−9^ M, and 10^−10^ M) of PFOS or PFBS with or without 1 nM dihydrotestosterone (DHT), with two biological replicates and six technical replicates. Treatments were repeated after two days. The effect of PFAS on cell viability was quantified after two days using the WST-1 cell proliferation assay as described [28,29,30,31]. Absorbance readings were measured at 450 nm using a Cytation 5 plate reader (BioTek, Winooski, VT, USA). Statistical analyses were performed using Graphpad Prism 8 software (GraphPad Software Inc., La Jolla, CA, USA). 

### 2.2. In Vivo Prostate Cancer Xenograft Model

Mouse experiments and protocols were approved by the University of Illinois at Urbana-Champaign (IACUC Protocol #20159), and National Institutes of Health standards for the use and care of animals were followed. RWPE-KR prostate cancer epithelial cell lines were used for the tumor xenograft study. Four-week-old athymic nude male mice (RRID:RGD_5508395) were obtained from Jackson Laboratory (stock no. 007850; Bar Harbor, ME, USA). After a week of acclimatization to test the synergy between PFAS and a high-fat diet (HFD), we compared carcinogenesis in mice fed an HFD to mice fed a control diet. Mice were fed ad libitum. As standard diets contain isoflavones with estrogenic activity that interfere with metabolic effects, we used F4031 diet (Bio-Serv, Flemington, NJ, USA) as control diet. Purified HFDs for our studies (F3282 diet, Bio-Serv, USA), meant to mimic the “Western diet”, are high in butterfat (~42% Kcal from fat) and polysaccharide. This diet is commonly used in metabolic syndrome studies [28,32,33].

Ten days after diets were initiated, 2 × 10^6^ RWPE-kRAS cells suspended in Matrigel were injected into the left and right flanks of mice (*N* = 8 mice/group) under anesthesia. In addition, silastic tubes packed with testosterone were implanted to provide the continuous testosterone needed for xenograft establishment and growth. Mice were administered PFOS by oral gavage seven days per week at 10 mg/kg. Food consumption and animal weights were monitored twice weekly. Tumor size measurements were obtained three times per week using digital calipers. Tumor volumes were calculated using formula V = 0.5 × length × width^2^ [34]. Animals were euthanized five weeks after the initial cancer cell line injection. Tumors, livers, prostates, and blood were harvested and either flash frozen or fixed in formalin for future staining.

### 2.3. OMICS-Based Metabolic Profiling

RWPE-Kras cells were seeded in growth media. The next day, cells were treated with a vehicle (Veh): 5 mL of 10^−8^ M PFOS or 5 mL of 10^−8^ M PFBS with or without 10^−9^ M DHT. Cell metabolites were extracted using a 1:2:1 mixture of acetonitrile, isopropanol, and water, respectively. Extracts were sent to the University of Illinois at Urbana-Champaign’s Metabolomics Core Facility to detect and quantify metabolites using gas chromatography mass spectroscopy (GC/MS). Metabolic profiles were obtained from an Agilent GC/MS system (Agilent 7890 gas chromatograph, Agilent 5975 MSD, and HP 7683B autosampler, Lexington, MA, USA). 

The spectra of all chromatogram peaks were evaluated using the AMDIS 2.71 and a custom-built database with 460 unique metabolites. All known artificial peaks were identified and removed before data mining. Individual metabolomic data sets for each treatment were separated and grouped into files to make comparisons between treatment conditions using Metaboanalyst software [35]. Sample class annotations consisted of Veh vs. PFOS, Veh vs. PFBS, Veh vs. DHT, DHT vs. DHT + PFOS, and DHT vs. DHT + PFBS. Files were uploaded to the Enrichment Analysis tool of MetaboAnalyst software version 5.0 (RRID:SCR_015539). Data were not normalized, transformed, or scaled but were compared to the SMPDB reference metabolome, which represents metabolite values from normal metabolic human pathways. The top 25 enriched metabolic pathways and associated metabolites were retrieved along with their p-values and enrichment ratios. Heatmaps were developed for each treatment group based on class averages using default settings for clustering, and the data were restricted to the top 25 metabolites using PLS-DA VIP.

For sequencing-based transcriptome analysis, RWPE-kRas xenograft tumors were used. Total RNA was isolated using Trizol reagent as per the manufacturer’s recommendations. cDNA libraries were prepared and sequencing reactions were performed by UIUC Sequencing core. Processing of data and analysis were performed as previously described [36,37,38,39,40,41]. Gene set enrichment analysis (GSEA) was performed to identify enriched gene set grouping as previously described [37,38,41]. 

### 2.4. Plate-Based Pyruvate and Acetyl Coa Assays

We prepared metabolite extracts from in vitro cell models and xenograft tumors to validate changes in pyruvate and acetyl-CoA levels using fluorescence-based plate assays (#MAK071 and #MAK039; Sigma, St. Louis, MO, USA). RWPE-kRAS cells were seeded in 10 cm plates at a density of 500,000 cells/plate and were treated with Veh (cell growth media), 5 mL of 10^−8^ M PFOS, or 5 mL of 10^−8^ M PFBS with or without 10^−9^ M DHT for 24 h. Pyruvate concentrations in the cells were detected by a Cytation 5 plate reader upon formation of fluorescent metabolite as pyruvate underwent oxidation by pyruvate oxidase. Pyruvate concentrations were reported in nmol/uL. 

For acetyl CoA measurement, the fluorescence produced from NADH and probe reaction coupled to conversion of acetyl-CoA to CoA was detected using a Cytation 5 plate reader. Each experiment was repeated twice with three technical replicates.

### 2.5. Western Blotting for Epigenetic Marker Assessment

RWPE-kRAS cells were seeded at a density of 500,000 cells/plate on 10 cm plates. Cells were treated with Veh (cell growth media), 5 mL of 10^−8^ M PFOS, or 5 mL of 10^−8^ M PFBS with or without 10^−9^ M DHT for 24 h. Cell lysates were collected in lysis buffer (0.5 M EDTA, 1 M TrisHCl pH 8.1, 10% SDS, 10% Empigen, ddH_2_O) with 1X complete protease inhibitor (Roche, Basel, Switzerland) and 1X phosphatase inhibitor (Thermo Scientific, Waltham, MA, USA). Cell lysates were sonicated and protein concentrations were determined by BCA assay (Thermo Scientific). Samples were boiled in SDS-containing loading buffer; each sample was run in 10% precast gels (BioRad, Hercules, CA, USA) and transferred to nitrocellulose membranes. Membranes were blocked in Blocking Buffer (Odyssey, Li-Cor, Lincoln, NE, USA). Target proteins were probed with acetyl histone antibody sampler kit (#9933, Cell Signaling, Danvers, MA, USA) (RRID: AB_10699455), tri-methyl histone antibody sampler kit (#9783, Cell Signaling) antibodies at 1:1000 dilution, and β-actin (SAB1305546, Sigma) (RRID: AB_2541177) antibody at 1:10,000 dilution. Secondary antibodies obtained from Odyssey were used at 1:10,000 dilution. Membranes were visualized using a LI-COR Odyssey CLx infrared imaging device and software.

## 3. Results

### 3.1. PFAS Exposure Increases Cell Proliferation in Malignant Prostate Cancer Cell Lines

**Figure 1:**
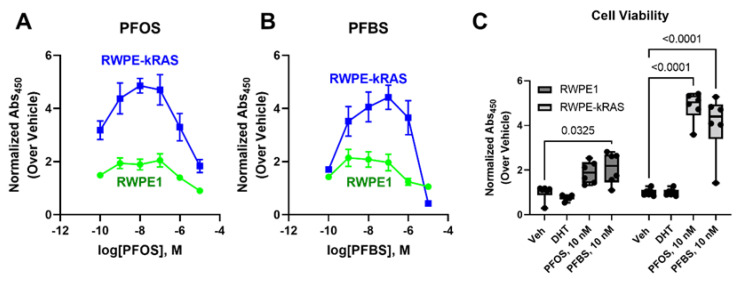
WST-1 assay shows that PFOS (**A**) or PFBS (**B**) exposure for 1 week increases cell viability of prostate benign (RWPE-1) and cancerous (RWPE-kRAS) cells (**C**). Comparison of cell viability of RWPE1 and RWPE-kRAS cells, when they are treated with Veh, 1 nM DHT, or 10 nM of PFAS.

To determine the impact of PFAS exposure on prostate cancer cells, we performed a cell viability assay using a benign human prostate cell line, RWPE-1, and a derivative cancerous cell line, RWPE-kRAS. Exposure to DHT had little effect whereas PFAS exposure enhanced cell viability at an environmentally relevant dose in both cell types (Figure 1). When benign and prostate cancer cells were exposed to varying concentrations of PFAS (10^−10^ M–10^−4^ M), they exhibited an inverted U-shaped dose–response curve. Cell proliferation increased at 10^−10^ PFOS (Figure 1A) and PFBS (Figure 1B) compared to vehicle and peaked at 10^−8^ M. RWPE-kRAS cells showed a more robust cell proliferation response compared to benign RWPE-1 cells, with the 10^−8^ M peak retained through the 10^−6^ M dose for both compounds. The aggressive RWPE-kRAS cells exhibited a significant 3.1-fold increase in cell proliferation when exposed to PFOS and a significant 5-fold increase in the cell viability when exposed to PFBS compared to Veh-treated cells. At higher doses, the stimulatory effects dissipated, declining below vehicle levels 10^−5^ M for both of the cell lines studied. 

### 3.2. Exposure to PFAS Increases RWPE-kRAS Xenograft Tumor Growth In Vivo

**Figure 2:**
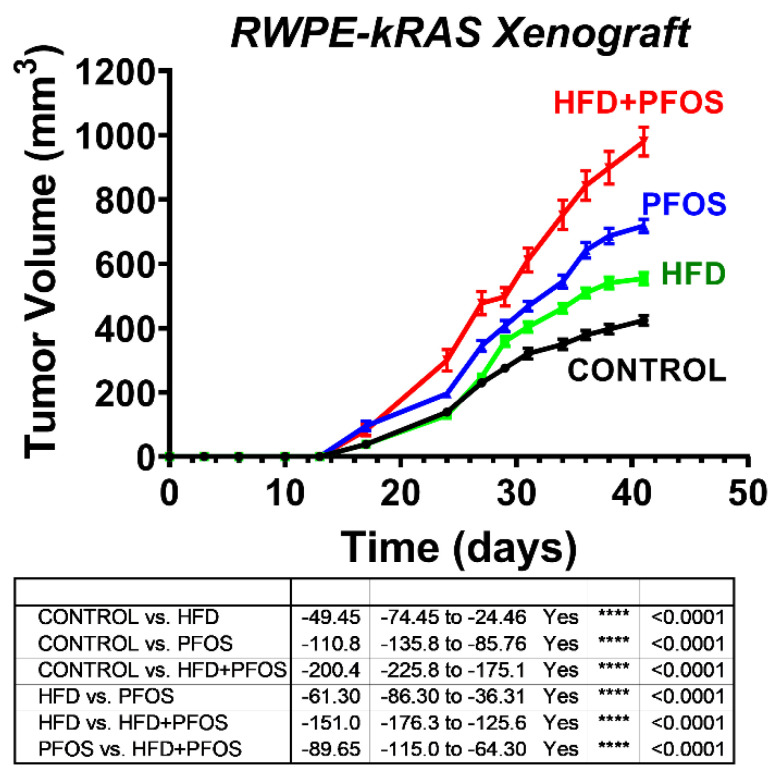
PFAS and an HFD synergize to increase prostate cancer xenograft growth. The 1 × 10^6^ RWPE-kRAS cells were injected subcutaneously in 4-week-old athymic nude male mice. Mice were fed an HFD or control diet and treated with 10 mg/kg oral PFOS or vehicle control 5 days/week for 40 days. Tumor volume was measured using electronic calipers three times/week. A two-way ANOVA model for the time dependent effects of treatments on tumor growth was fitted. When the change is significant, Tukey’s multiple comparison test was employed.

An HFD may contribute to prostate cancer progression by shifting the prostate metabolome to a pro-cancerous state [26,27]. These actions are mediated through PPARα, the receptor targeted by PFAS, providing the potential for synergistic tumor promotion. To evaluate the effects of PFAS exposure, we generated a xenograft tumor growth model in nude immunocompromised mice by injecting RWPE-kRAS cells that were fed either a control diet or an HFD and exposed to daily PFOS or control gavage. We selected these cells since PFAS treatments had a more robust effect on cell viability compared to that of RWPE1 cells. At 40 days post-injection, we observed an increase in ectopic tumor volume with PFOS exposure or an HFD alone; however, the fastest rate of growth was observed in mice exposed to PFOS and fed an HFD, indicating a synergistic response (Figure 2). 

### 3.3. PFAS Treatment Change Metabolic Phenotype of Prostate Cancer Cells

**Figure 3:**
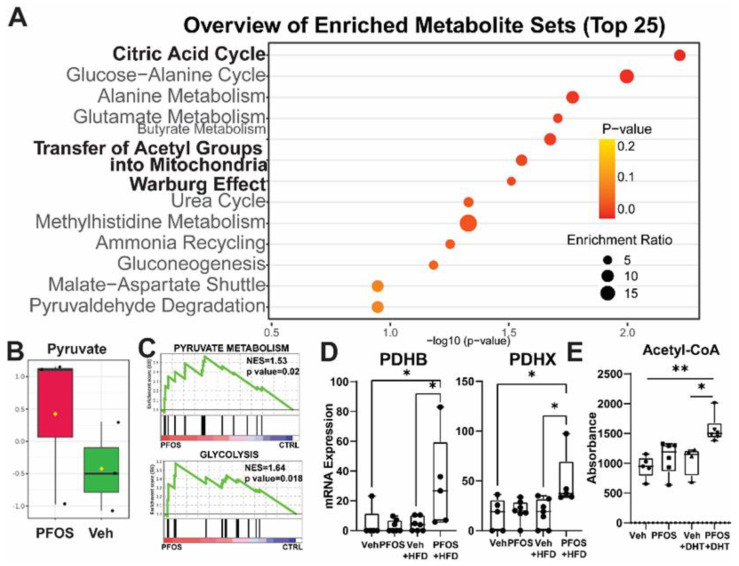
PFAS treatment increases pyruvate and acetyl-CoA levels in RWPE-kRAS cells. (**A**) PFOS-induced metabolites in RWPE-kRAS cells identified by GC/MS analysis. (**B**) Pyruvate levels from (**A**), (**C**) GSEA of PFOS + HFD-induced genes in RWPE-kRAS xenografts identified by RNA-seq. (**D**) mRNA expression of PDHB and PDHX, components of PDC, were increased with PFOS and a high-fat diet (HFD) in RWPE-kRAS xenografts. (**E**) Acetyl-CoA levels in PFOS-treated RWPE-kRAS cells (10 nM PFOS ± DHT, 24 h) using a fluorescence-based assay. * *p* < 0.05, ** *p* < 0.01.

Previous work using transgenic mouse models of Myc-induced prostate cancer shows that an HFD increases one-carbon metabolism and is associated with changes in histone methylation, further increasing Myc activity in prostate tumors [26]. However, the impact of environmental exposures on prostate cancer cell metabolic wiring is unknown. Since we observed synergy between PFOS exposure and an HFD in increasing the RWPE-kRAS tumor burden, and an increase in RWPE-kRAS cell viability with PFOS treatment, we performed multiple -omics analyses to examine metabolic changes in these cells (Figure 3). The prostate cancer cells analyzed exhibited an increased proliferative response with PFAS exposure in the presence of DHT; thus, we hypothesized that cell-proliferative energetic pathways would be upregulated in the cell metabolome. To test this, we analyzed metabolites that changed in response to PFOS treatment using GC/MS analysis of RWPE-kRAS cell extracts (Figures S1 and S2). PFOS treatment increased metabolites associated with glucose metabolism via the Warburg effect, involving the transfer of acetyl groups into mitochondria and the citric acid cycle (Figure 3A), particularly pyruvate (Figure 3B). 

To determine whether the observed increase in pyruvate production was due to increased expression of enzymes in the glycolytic pathway, we next performed RNA-seq using tumors from Figure 2. PFOS exposure in animals that are fed HFD-upregulated gene sets is related to prostate carcinogenesis (Supplementary Tables S1–S9). GSEA identified genes involved in pyruvate metabolism and glycolysis pathways as significantly upregulated by PFOS exposure in tumors from mice fed an HFD (Figure 3C, Supplementary Table S2). In particular, components of the pyruvate dehydrogenase complex (PDC), responsible for acetyl-CoA production from pyruvate, were increased with PFOS exposure in tumors from mice fed an HFD (Figure 3D). Consistent with these results, acetyl-CoA was increased in RWPE-kRAS cells with increased cell viability (Figure 3E). These results from transformed prostate cells indicate that PFAS exposure increases pyruvate and acetyl-CoA production. 

We observed that PFAS treatment upregulated threonine and 2-oxobutanoate degradation (4.757-fold, *n* = 3, *p* = 0.000903), phosphatidylethanolamine biosynthesis (4.057-fold, *n* = 3, *p* = 0.0143), homocysteine degradation (4.036-fold, *n* = 3, *p* = 0.0144), and lysine degradation (3.403-fold, *p* =0.0165), all pathways involved in mitochondrial dependence, citric acid cycle regulation, and the pentose phosphate pathway [42,43] (Figure S1). Interestingly, biotin metabolism, which plays an important role in acetyl-CoA carboxylase function as a prosthetic group, also was significantly upregulated (4.039-fold, *n* = 3, *p* = 0.0148). These data reinforce our hypothesis that PFAS treatment affects acetyl-CoA metabolism (Figure S1). 

### 3.4. PFAS Treatment Increases PPAR Signaling and Histone Acetylation in Prostate Cancer Cells

**Figure 4:**
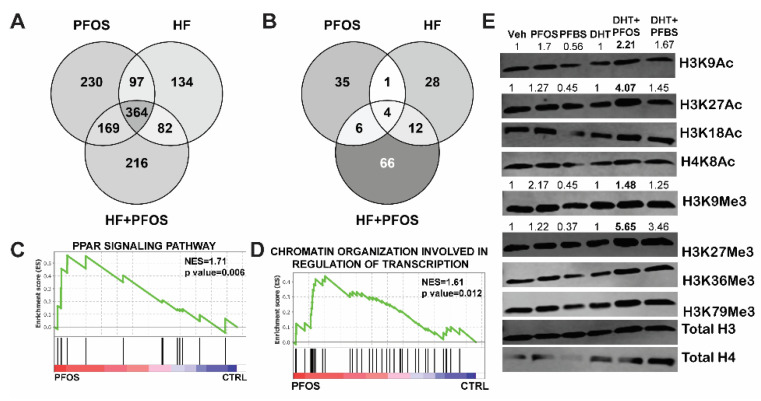
Venn diagram analysis of up- (**A**) or downregulated (**B**) genes in different treatment groups compared to the control group. (**C**) RNA-seq data indicate that PFAS exposure combined with a high-fat diet increased PPAR signaling in RWPE-kRAS xenografts. (**D**) RNA-seq data also indicated epigenetic regulation of transcription-associated genes in RWPE-kRAS xenografts. (**E**) Western blots demonstrating PFAS exposure increased histone acetyl markers in RWPE-kRAS cells. Numbers above bands indicate quantitation of signal for marks in samples that are treated with DHT + PFAS that change over DHT treatments.

To interrogate transcriptional changes in prostate tumors exposed to PFAS, we further analyzed the RNA-seq data from RWPE-kRAS xenografts. The comparison of the number of genes up- (Figure 4A) or down- (Figure 4B) regulated by PFOS, HFD, or HFD + PFOS showed that each treatment resulted in distinct gene regulation patterns compared to tumors from the control treatment group. The PFOS exposure combined with an HFD, which increased tumor growth, induced transcriptomic changes in PPARα-target genes and genes involved in chromatin organization that, in turn, regulate transcription (Figure 4C,D, Supplementary Table S3). In a transgenic MYC model of prostate carcinogenesis, an HFD induced changes in H4K20 methylation and impacted expression of Myc target genes [26]. Consistent with these results as well as earlier changes identified in pyruvate and acetyl-CoA synthesis-related metabolites, we examined a range of histone acetylation and methylation markers in RWPE-kRAS cells exposed to PFOS and identified significant increases in these marks (Figure 4E). These data support a fundamental role of PPAR signaling and epigenetic changes in prostate cancer xenograft response to a combination of PFAS and other procarcinogenic stimuli. 

## 4. Discussion

To explore the potential role of PFAS in prostate cancer, we characterized the effect of PFAS on prostate benign and cancer cell lines by evaluating cell proliferation, metabolomics, and metabolite profiling as well as growth, transcriptomics, and metabolomics using in vivo xenograft models. Our findings indicate that PFAS exposure increases in vitro prostate benign and cancer cell proliferation nearly three-fold and increases the rate of tumor growth in mouse models. Using metabolite profiling assays, we found that PFAS shifts the cellular energetics of prostate cancer cells, moving the cells to a more energetically efficient and mitochondria-dependent state by enhancing oxidative phosphorylation and upregulating the pentose phosphate pathway and citric acid cycle. 

Epidemiology studies show that prostate cancer risk and mortality increase with PFAS exposure [22,23,24] and obesity [25,44]. Despite this evidence, mechanistic data on the molecular underpinnings of PFAS chemicals in the prostate have been limited. We show that an HFD and PFAS exposure synergize to increase prostate cancer xenograft growth in mice. Further, PFAS treatment increases glucose metabolism and pyruvate production in these tumors. It is well established that metabolic adaptations in prostate cancer alter the epigenetic landscape, in part due to changes in substrate availability for epigenetic enzymes. There is no evidence of genotoxicity associated with PFAS [45,46,47], suggesting they elicit effects without causing direct DNA mutations. One plausible means through which PFAS may exert effects is epigenetic and transcriptomic alterations. Associations between PFAS exposures and altered methylation, either genome-wide or at specific histone loci, are described by several laboratories [48,49,50,51]. PFAS exposure is also associated with lower global DNA methylation in neonates [52,53,54].

PPARs are transcription factors involved in regulating metabolic processes, and an HFD impacts hepatic cells through PPARα activation [55]. We previously determined that metabolites associated with obesity activate PPARα signaling to modulate ERα activity in breast cancer cells [36], while PFAS activate PPARα to affect metabolism and the immune system [56]. Structurally, PFAS resemble free fatty acids and bind to the same sites on serum proteins [57]. Further, PPARα signaling plays a central role in PFOA/PFOS-induced liver and kidney carcinogenesis [58,59]. In addition, epigenetic marks dictate the activity of PPARα, a critical transcription factor in PFAS-associated carcinogenesis [58,59] liganded by both PFAS [57] and metabolites associated with an HFD [55]. However, this pathway has not been examined in the context of prostate cancer. Our data suggest that PFAS exposure has a synergy with an HFD to activate PPARα altering the cell metabolome, which shifts carcinogenic risk in normal cells while driving cancer progression in prostate cancer cells.

The present findings show that metabolic alterations from an HFD combined with PFAS exposure play a significant role in prostate tumor growth and progression. Together, our study suggests that alterations in cell metabolism downstream of PPARα activation by PFAS and HFDs may underpin the increased prostate cancer risk observed in PFAS-exposed men.

## 5. Conclusions

Our findings suggest that PFAS play a role in prostate cancer development and tumor progression. Further investigation of the specific types of cancers associated with PPAR activation is needed to determine the link between specific PFAS and cancers and to elucidate the underlying mechanism of PFAS action in cancer development. Understanding the activation on PPARα, β/δ, and γ by different types of PFAS will provide insight on toxicity levels and how PFAS-associated cancers are initiated. Additional research is needed to pinpoint specific alternative substances that can be used as PFAS substitutes. Research is necessary to protect consumer health, especially among occupations with high exposures such as firefighters. Researchers and manufacturers must work together to develop alternative substances that do not pose a substantial risk to health.

## Data Availability

RNA-seq data are available in GEO under GSE185183.

## Supplementary Materials

The following are available online at https://www.mdpi.com/article/10.3390/nu13113902/s1, Figure S1: PFAS exposure upregulates mitochondrial dependence (citric acid cycle, PPP), and altered amino acid metabolism (serine & lysine). Metabolic pathways that were upregulated in the PFAS-treated group compared to a non-treated control group were cross referenced and pathways that were upregulated in the PFAS group were listed based on enrichment ratio, Figure S2: Pathway enrichment analysis of metabolites regulated in tumors., Table S1: GSEA related to prostate associated gene sets of HFD vs. HFD + PFOS group, Table S2: GSEA related to metabolism associated gene sets of HFD vs. HFD + PFOS group, Table S3: GSEA related to PPAR targets associated gene sets of HFD vs. HFD + PFOS group, Table S4: GSEA related to prostate associated gene sets of HFD vs. ctrl group, Table S5: GSEA related to metabolism associated gene sets of HFD vs. ctrl group, Table S6: GSEA related to PPAR targets associated gene sets of HFD vs. ctrl group, Table S7: GSEA related to prostate associated gene sets of PFOS vs. ctrl group, Table S8: GSEA related to metabolism associated gene sets of PFOS vs. ctrl group, Table S9: GSEA related to PPAR targets associated gene sets of PFOS vs. ctrl group.
