# Supplementary material for: Per- and Polyfluoroalkyl Substance Exposure Combined with High-Fat Diet Supports Prostate Cancer Progression
Source: Nutrients. 2021 Oct 30;13(11):3902. doi: 10.3390/nu13113902 (PMC8623692; doi:10.3390/nu13113902)
Supplement: Supplementary file 1 [file nutrients-13-03902-s001.zip › nutrients-1415459-supplementary.pdf]

A

Metabolism	P Value	Enrichment Ratio
Threonine & 2-Oxobutanoate Degradation	9.0355E-4	4.757
Phosphatidylethanolamine Biosynthesis	0.014279	4.057
Homocysteine Degradation	0.014407	4.036
Selenoamino Acid Metabolism	0.014687	3.620
Biotin Metabolism	0.014849	4.039
Lysine Degradation	0.016517	3.403
Methionine Metabolism	0.016937	3.106
Glycine & Serine Metabolism	0.023257	2.599
Catecholamine Biosynthesis	0.038154	3.494
Thyroid Hormone Synthesis	0.038154	3.494
Carnitine Synthesis	0.24275	1.494
Ammonia Recycling	0.25056	1.533

B

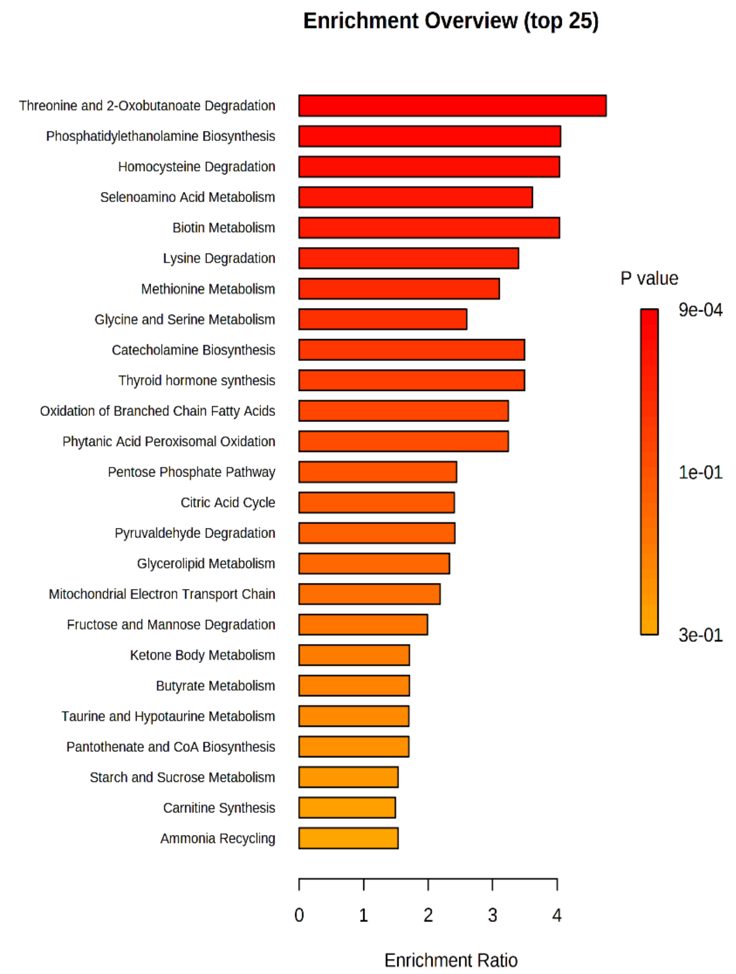

Figure S1. PFAS exposure upregulates mitochondrial dependence (citric acid cycle, PPP), and altered amino acid metabolism (serine & lysine) . Metabolic pathways that were upregulated in the PFAS-treated group compared to a non-treated control group were cross referenced and pathways that were upregulated in the PFAS group were listed based on enrichment ratio.

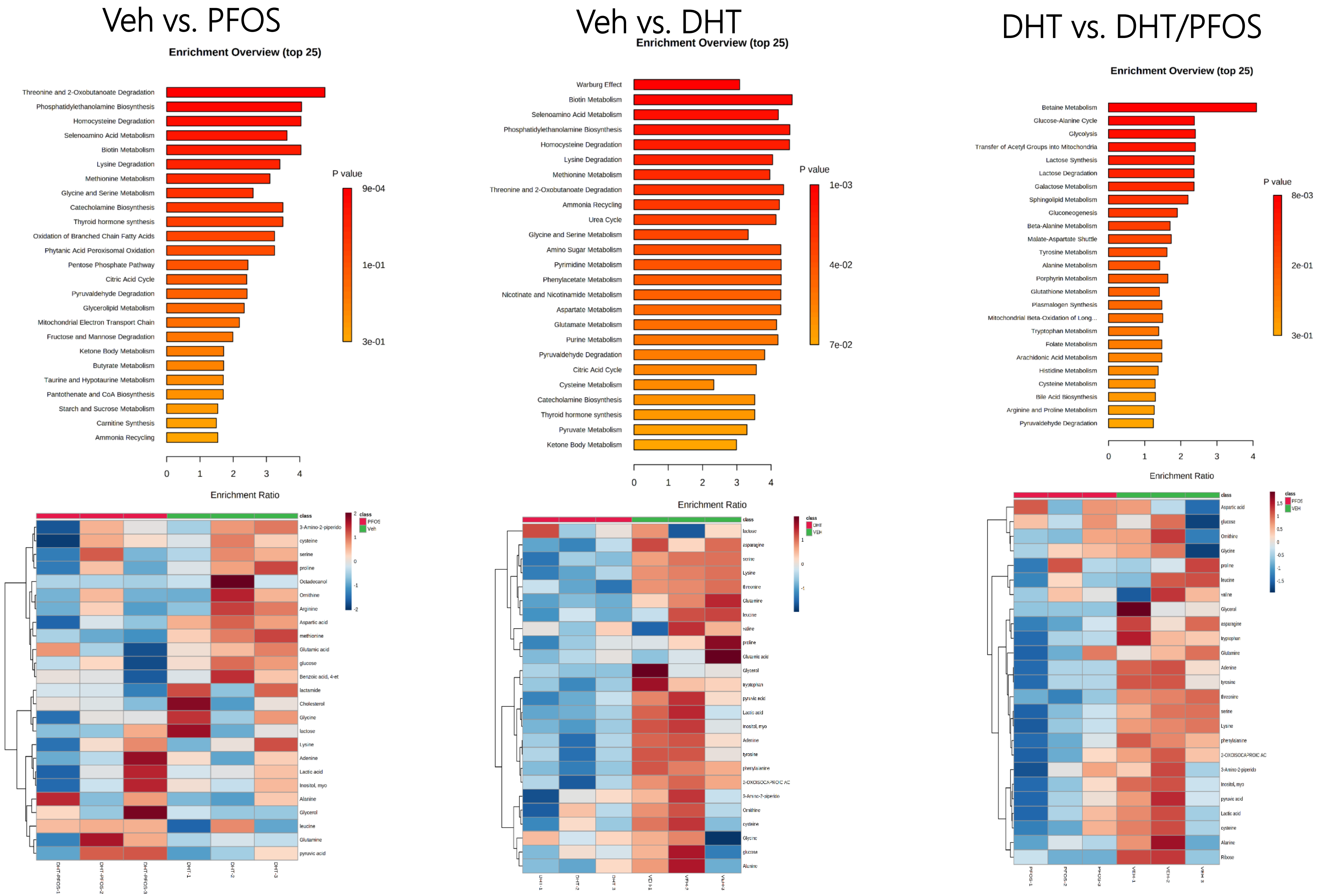

Veh vs. PFOS
Enrichment Overview (top 25)
Threonine and 2-Oxobutanoate Degradation
Phosphatidylethanolamine Biosynthesis
Homocysteine Degradation
Selenoamino Acid Metabolism
Biotin Metabolism
Lysine Degradation
Methionine Metabolism
Glycine and Serine Metabolism
Catecholamine Biosynthesis
Thyroid hormone synthesis
Oxidation of Branched Chain Fatty Acids
Phytanic Acid Peroxisomal Oxidation
Pentose Phosphate Pathway
Citric Acid Cycle
Pyruvaldehyde Degradation
Glycerolipid Metabolism
Mitochondrial Electron Transport Chain
Fructose and Mannose Degradation
Ketone Body Metabolism
Butyrate Metabolism
Taurine and Hypotaurine Metabolism
Pantothenate and CoA Biosynthesis
Starch and Sucrose Metabolism
Carnitine Synthesis
Ammonia Recycling
P value
9e-04
1e-01
3e-01
0
1
2
3
4
Enrichment Ratio
class
PFOS
Veh
3-Amino-2-piperido
cysteine
serine
proline
Octadecanol
Ornithine
Arginine
Aspartic acid
methionine
Glutamic acid
glucose
Benzoic acid, 4-et
lactamide
Cholesterol
Glycine
lactose
Lysine
Adenine
Lactic acid
Inositol, myo
Alanine
Glycerol
leucine
Glutamine
pyruvic acid
DHT-PFOS-1
DHT-PFOS-2
DHT-PFOS-3
DHT-1
DHT-2
DHT-3
Veh vs. DHT
Enrichment Overview (top 25)
Warburg Effect
Biotin Metabolism
Selenoamino Acid Metabolism
Phosphatidylethanolamine Biosynthesis
Homocysteine Degradation
Lysine Degradation
Methionine Metabolism
Threonine and 2-Oxobutanoate Degradation
Ammonia Recycling
Urea Cycle
Glycine and Serine Metabolism
Amino Sugar Metabolism
Pyrimidine Metabolism
Phenylacetate Metabolism
Nicotinate and Nicotinamide Metabolism
Aspartate Metabolism
Glutamate Metabolism
Purine Metabolism
Pyruvaldehyde Degradation
Citric Acid Cycle
Cysteine Metabolism
Catecholamine Biosynthesis
Thyroid hormone synthesis
Pyruvate Metabolism
Ketone Body Metabolism
P value
1e-03
4e-02
7e-02
0
1
2
3
4
Enrichment Ratio
class
DHT
VEH
DHT vs. DHT/PFOS
Enrichment Overview (top 25)
Betaine Metabolism
Glucose-Alanine Cycle
Glycolysis
Transfer of Acetyl Groups into Mitochondria
Lactose Synthesis
Lactose Degradation
Galactose Metabolism
Sphingolipid Metabolism
Gluconeogenesis
Beta-Alanine Metabolism
Malate-Aspartate Shuttle
Tyrosine Metabolism
Alanine Metabolism
Porphyrin Metabolism
Glutathione Metabolism
Plasmalogen Synthesis
Mitochondrial Beta-Oxidation of Long...
Tryptophan Metabolism
Folate Metabolism
Arachidonic Acid Metabolism
Histidine Metabolism
Cysteine Metabolism
Bile Acid Biosynthesis
Arginine and Proline Metabolism
Pyruvaldehyde Degradation
P value
8e-03
2e-01
3e-01
0
1
2
3
4
Enrichment Ratio
class
PFOS
VEH

Figure S2

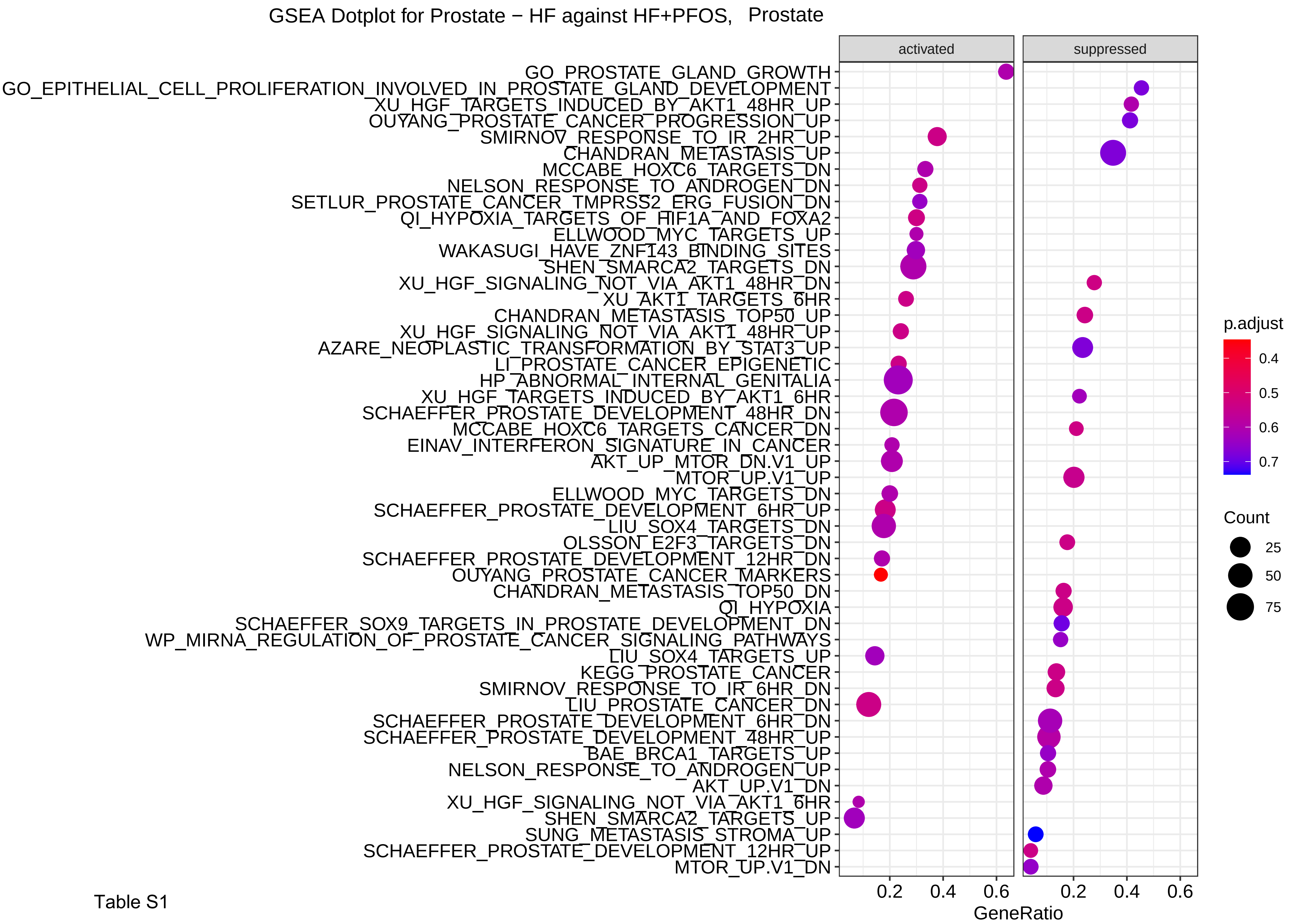
GSEA Dotplot for Prostate − HF against HF+PFOS, Prostate
activated
suppressed
GO_PROSTATE_GLAND_GROWTH
GO_EPITHELIAL_CELL_PROLIFERATION_INVOLVED_IN_PROSTATE_GLAND_DEVELOPMENT
XU_HGF_TARGETS_INDUCED_BY_AKT1_48HR_UP
OUYANG_PROSTATE_CANCER_PROGRESSION_UP
SMIRNOV_RESPONSE_TO_IR_2HR_UP
CHANDRAN_METASTASIS_UP
MCCABE_HOXC6_TARGETS_DN
NELSON_RESPONSE_TO_ANDROGEN_DN
SETLUR_PROSTATE_CANCER_TMPRSS2_ERG_FUSION_DN
QI_HYPOXIA_TARGETS_OF_HIF1A_AND_FOXA2
ELLWOOD_MYC_TARGETS_UP
WAKASUGI_HAVE_ZNF143_BINDING_SITES
SHEN_SMARCA2_TARGETS_DN
XU_HGF_SIGNALING_NOT_VIA_AKT1_48HR_DN
XU_AKT1_TARGETS_6HR
CHANDRAN_METASTASIS_TOP50_UP
XU_HGF_SIGNALING_NOT_VIA_AKT1_48HR_UP
AZARE_NEOPLASTIC_TRANSFORMATION_BY_STAT3_UP
LI_PROSTATE_CANCER_EPIGENETIC
HP_ABNORMAL_INTERNAL_GENITALIA
XU_HGF_TARGETS_INDUCED_BY_AKT1_6HR
SCHAEFFER_PROSTATE_DEVELOPMENT_48HR_DN
MCCABE_HOXC6_TARGETS_CANCER_DN
EINAV_INTERFERON_SIGNATURE_IN_CANCER
AKT_UP_MTOR_DN.V1_UP
MTOR_UP.V1_UP
ELLWOOD_MYC_TARGETS_DN
SCHAEFFER_PROSTATE_DEVELOPMENT_6HR_UP
LIU_SOX4_TARGETS_DN
OLSSON_E2F3_TARGETS_DN
SCHAEFFER_PROSTATE_DEVELOPMENT_12HR_DN
OUYANG_PROSTATE_CANCER_MARKERS
CHANDRAN_METASTASIS_TOP50_DN
QI_HYPOXIA
SCHAEFFER_SOX9_TARGETS_IN_PROSTATE_DEVELOPMENT_DN
WP_MIRNA_REGULATION_OF_PROSTATE_CANCER_SIGNALING_PATHWAYS
LIU_SOX4_TARGETS_UP
KEGG_PROSTATE_CANCER
SMIRNOV_RESPONSE_TO_IR_6HR_DN
LIU_PROSTATE_CANCER_DN
SCHAEFFER_PROSTATE_DEVELOPMENT_6HR_DN
SCHAEFFER_PROSTATE_DEVELOPMENT_48HR_UP
BAE_BRCA1_TARGETS_UP
NELSON_RESPONSE_TO_ANDROGEN_UP
AKT_UP.V1_DN
XU_HGF_SIGNALING_NOT_VIA_AKT1_6HR
SHEN_SMARCA2_TARGETS_UP
SUNG_METASTASIS_STROMA_UP
SCHAEFFER_PROSTATE_DEVELOPMENT_12HR_UP
MTOR_UP.V1_DN
0.2
0.4
0.6
0.2
0.4
0.6
GeneRatio
p.adjust
0.4
0.5
0.6
0.7
Count
25
50
75

Table S1

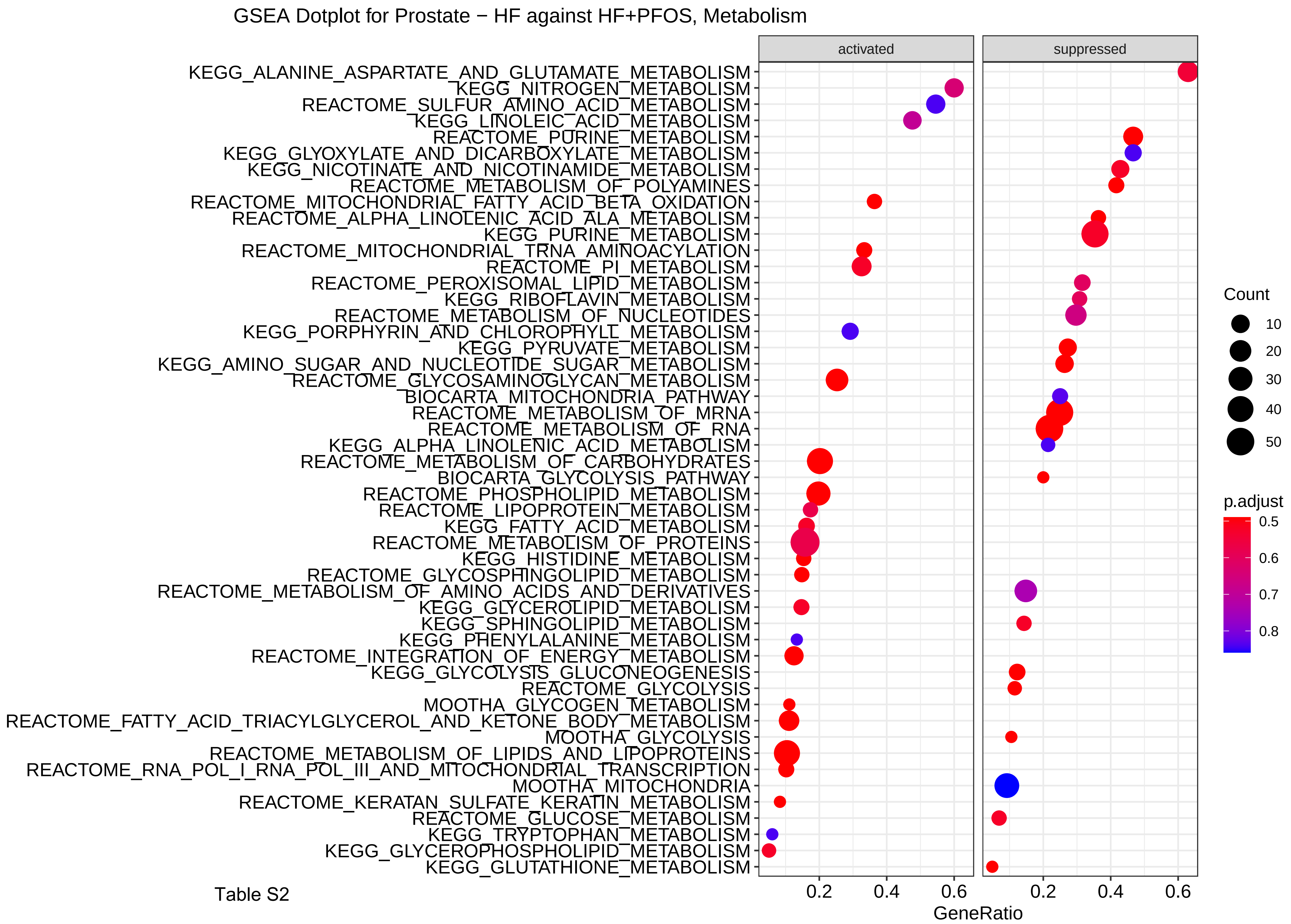
GSEA Dotplot for Prostate − HF against HF+PFOS, Metabolism
activated
suppressed
KEGG_ALANINE_ASPARTATE_AND_GLUTAMATE_METABOLISM
KEGG_NITROGEN_METABOLISM
REACTOME_SULFUR_AMINO_ACID_METABOLISM
KEGG_LINOLEIC_ACID_METABOLISM
REACTOME_PURINE_METABOLISM
KEGG_GLYOXYLATE_AND_DICARBOXYLATE_METABOLISM
KEGG_NICOTINATE_AND_NICOTINAMIDE_METABOLISM
REACTOME_METABOLISM_OF_POLYAMINES
REACTOME_MITOCHONDRIAL_FATTY_ACID_BETA_OXIDATION
REACTOME_ALPHA_LINOLENIC_ACID_ALA_METABOLISM
KEGG_PURINE_METABOLISM
REACTOME_MITOCHONDRIAL_TRNA_AMINOACYLATION
REACTOME_PI_METABOLISM
REACTOME_PEROXISOMAL_LIPID_METABOLISM
KEGG_RIBOFLAVIN_METABOLISM
REACTOME_METABOLISM_OF_NUCLEOTIDES
KEGG_PORPHYRIN_AND_CHLOROPHYLL_METABOLISM
KEGG_PYRUVATE_METABOLISM
KEGG_AMINO_SUGAR_AND_NUCLEOTIDE_SUGAR_METABOLISM
REACTOME_GLYCOSAMINOGLYCAN_METABOLISM
BIOCARTA_MITOCHONDRIA_PATHWAY
REACTOME_METABOLISM_OF_MRNA
REACTOME_METABOLISM_OF_RNA
KEGG_ALPHA_LINOLENIC_ACID_METABOLISM
REACTOME_METABOLISM_OF_CARBOHYDRATES
BIOCARTA_GLYCOLYSIS_PATHWAY
REACTOME_PHOSPHOLIPID_METABOLISM
REACTOME_LIPOPROTEIN_METABOLISM
KEGG_FATTY_ACID_METABOLISM
REACTOME_METABOLISM_OF_PROTEINS
KEGG_HISTIDINE_METABOLISM
REACTOME_GLYCOSPHINGOLIPID_METABOLISM
REACTOME_METABOLISM_OF_AMINO_ACIDS_AND_DERIVATIVES
KEGG_GLYCEROLIPID_METABOLISM
KEGG_SPHINGOLIPID_METABOLISM
KEGG_PHENYLALANINE_METABOLISM
REACTOME_INTEGRATION_OF_ENERGY_METABOLISM
KEGG_GLYCOLYSIS_GLUCONEOGENESIS
REACTOME_GLYCOLYSIS
MOOTHA_GLYCOGEN_METABOLISM
REACTOME_FATTY_ACID_TRIACYLGLYCEROL_AND_KETONE_BODY_METABOLISM
MOOTHA_GLYCOLYSIS
REACTOME_METABOLISM_OF_LIPIDS_AND_LIPOPROTEINS
REACTOME_RNA_POL_I_RNA_POL_III_AND_MITOCHONDRIAL_TRANSCRIPTION
MOOTHA_MITOCHONDRIA
REACTOME_KERATAN_SULFATE_KERATIN_METABOLISM
REACTOME_GLUCOSE_METABOLISM
KEGG_TRYPTOPHAN_METABOLISM
KEGG_GLYCEROPHOSPHOLIPID_METABOLISM
KEGG_GLUTATHIONE_METABOLISM
0.2
0.4
0.6
0.2
0.4
0.6
GeneRatio
Count
10
20
30
40
50
p.adjust
0.5
0.6
0.7
0.8

Table S2

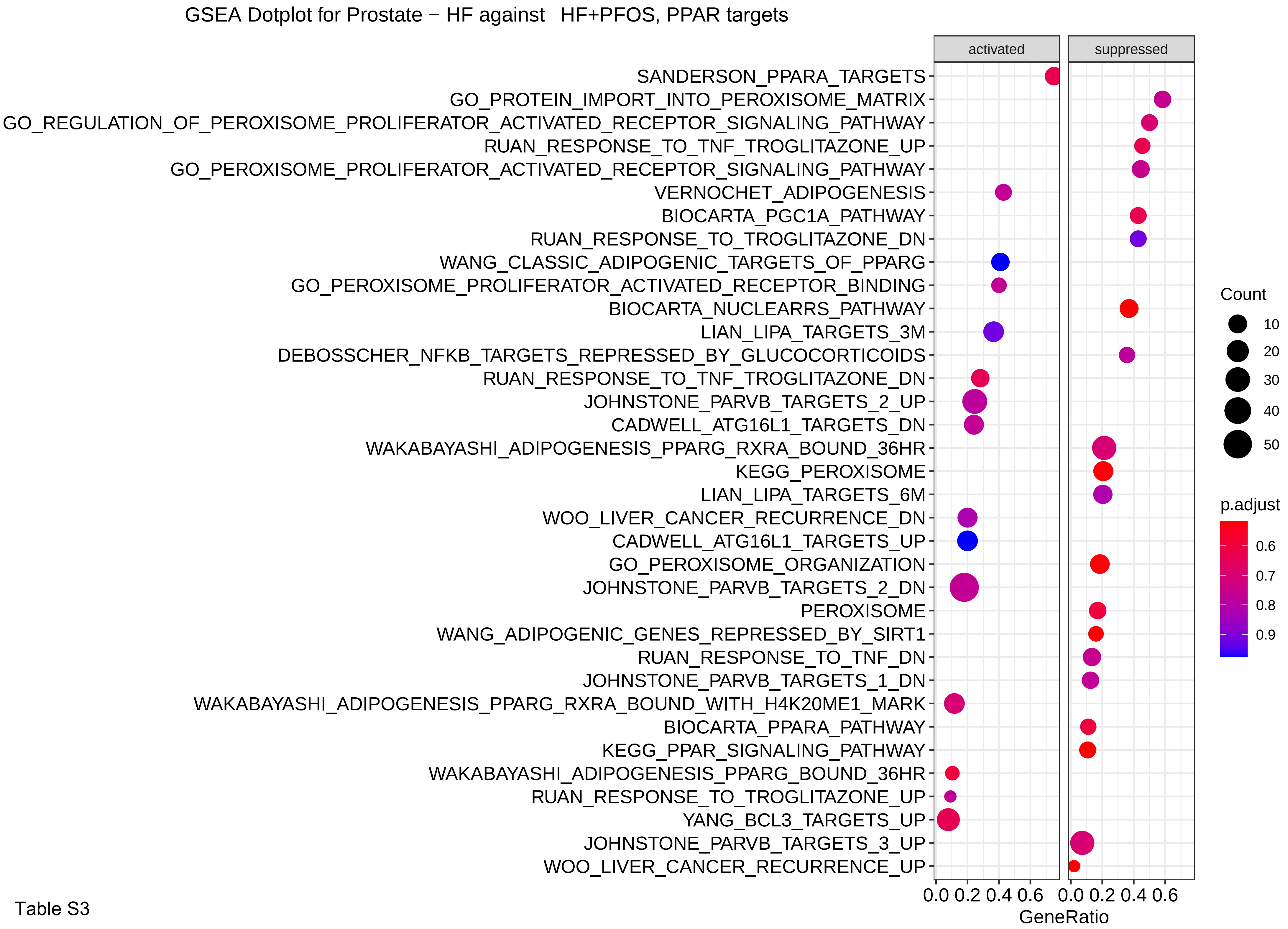
GSEA Dotplot for Prostate − HF against HF+PFOS, PPAR targets
activated
suppressed
SANDERSON_PPARA_TARGETS
GO_PROTEIN_IMPORT_INTO_PEROXISOME_MATRIX
GO_REGULATION_OF_PEROXISOME_PROLIFERATOR_ACTIVATED_RECEPTOR_SIGNALING_PATHWAY
RUAN_RESPONSE_TO_TNF_TROGLITAZONE_UP
GO_PEROXISOME_PROLIFERATOR_ACTIVATED_RECEPTOR_SIGNALING_PATHWAY
VERNOCHET_ADIPOGENESIS
BIOCARTA_PGC1A_PATHWAY
RUAN_RESPONSE_TO_TROGLITAZONE_DN
WANG_CLASSIC_ADIPOGENIC_TARGETS_OF_PPARG
GO_PEROXISOME_PROLIFERATOR_ACTIVATED_RECEPTOR_BINDING
BIOCARTA_NUCLEARRS_PATHWAY
LIAN_LIPA_TARGETS_3M
DEBOSSCHER_NFKB_TARGETS_REPRESSED_BY_GLUCOCORTICOIDS
RUAN_RESPONSE_TO_TNF_TROGLITAZONE_DN
JOHNSTONE_PARVB_TARGETS_2_UP
CADWELL_ATG16L1_TARGETS_DN
WAKABAYASHI_ADIPOGENESIS_PPARG_RXRA_BOUND_36HR
KEGG_PEROXISOME
LIAN_LIPA_TARGETS_6M
WOO_LIVER_CANCER_RECURRENCE_DN
CADWELL_ATG16L1_TARGETS_UP
GO_PEROXISOME_ORGANIZATION
JOHNSTONE_PARVB_TARGETS_2_DN
PEROXISOME
WANG_ADIPOGENIC_GENES_REPRESSED_BY_SIRT1
RUAN_RESPONSE_TO_TNF_DN
JOHNSTONE_PARVB_TARGETS_1_DN
WAKABAYASHI_ADIPOGENESIS_PPARG_RXRA_BOUND_WITH_H4K20ME1_MARK
BIOCARTA_PPARA_PATHWAY
KEGG_PPAR_SIGNALING_PATHWAY
WAKABAYASHI_ADIPOGENESIS_PPARG_BOUND_36HR
RUAN_RESPONSE_TO_TROGLITAZONE_UP
YANG_BCL3_TARGETS_UP
JOHNSTONE_PARVB_TARGETS_3_UP
WOO_LIVER_CANCER_RECURRENCE_UP
0.0 0.2 0.4 0.6
0.0 0.2 0.4 0.6
GeneRatio
Count
10
20
30
40
50
p.adjust
0.6
0.7
0.8
0.9

Table S3

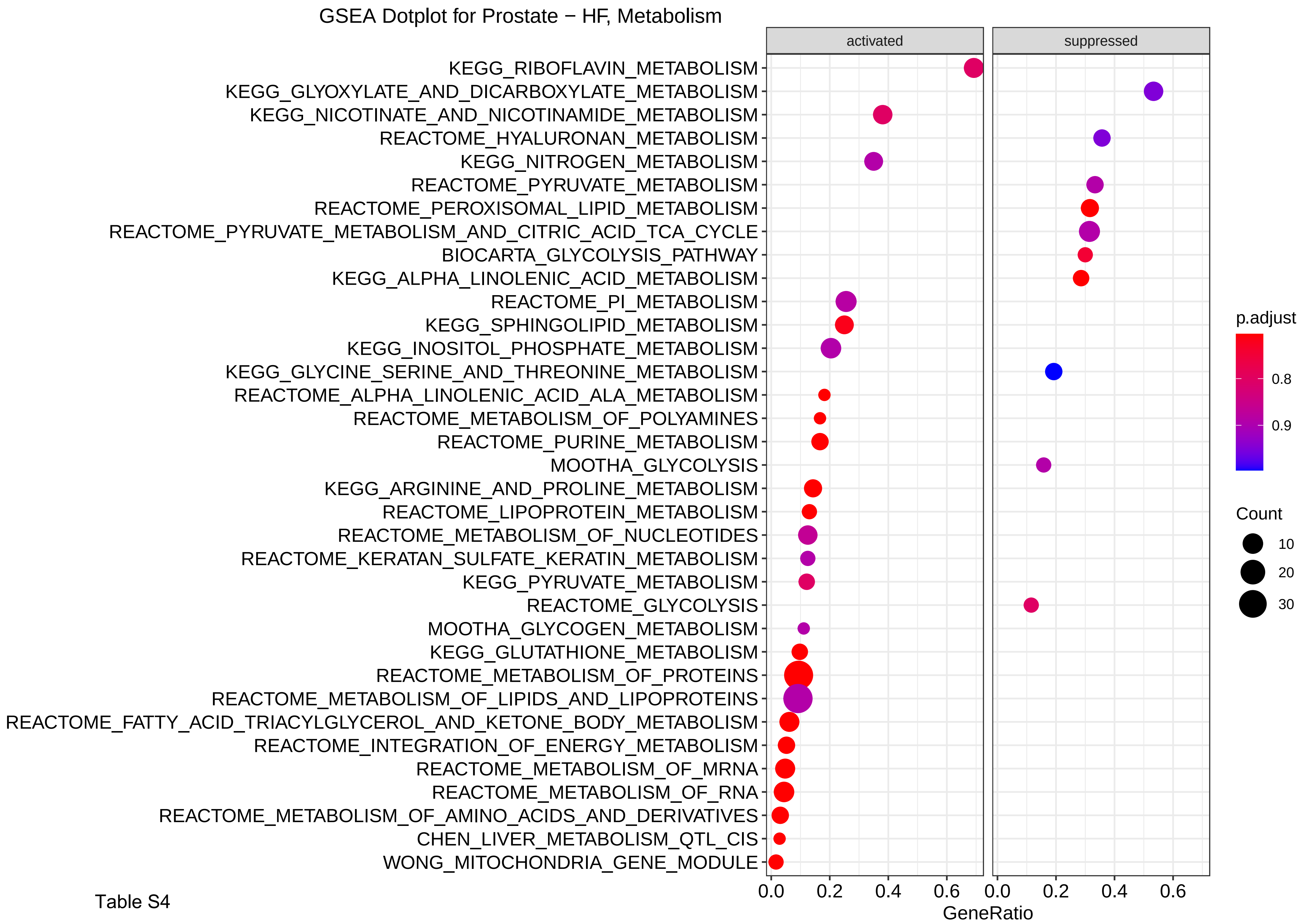
GSEA Dotplot for Prostate − HF, Metabolism
activated
suppressed
KEGG_RIBOFLAVIN_METABOLISM
KEGG_GLYOXYLATE_AND_DICARBOXYLATE_METABOLISM
KEGG_NICOTINATE_AND_NICOTINAMIDE_METABOLISM
REACTOME_HYALURONAN_METABOLISM
KEGG_NITROGEN_METABOLISM
REACTOME_PYRUVATE_METABOLISM
REACTOME_PEROXISOMAL_LIPID_METABOLISM
REACTOME_PYRUVATE_METABOLISM_AND_CITRIC_ACID_TCA_CYCLE
BIOCARTA_GLYCOLYSIS_PATHWAY
KEGG_ALPHA_LINOLENIC_ACID_METABOLISM
REACTOME_PI_METABOLISM
KEGG_SPHINGOLIPID_METABOLISM
KEGG_INOSITOL_PHOSPHATE_METABOLISM
KEGG_GLYCINE_SERINE_AND_THREONINE_METABOLISM
REACTOME_ALPHA_LINOLENIC_ACID_ALA_METABOLISM
REACTOME_METABOLISM_OF_POLYAMINES
REACTOME_PURINE_METABOLISM
MOOTHA_GLYCOLYSIS
KEGG_ARGININE_AND_PROLINE_METABOLISM
REACTOME_LIPOPROTEIN_METABOLISM
REACTOME_METABOLISM_OF_NUCLEOTIDES
REACTOME_KERATAN_SULFATE_KERATIN_METABOLISM
KEGG_PYRUVATE_METABOLISM
REACTOME_GLYCOLYSIS
MOOTHA_GLYCOGEN_METABOLISM
KEGG_GLUTATHIONE_METABOLISM
REACTOME_METABOLISM_OF_PROTEINS
REACTOME_METABOLISM_OF_LIPIDS_AND_LIPOPROTEINS
REACTOME_FATTY_ACID_TRIACYLGLYCEROL_AND_KETONE_BODY_METABOLISM
REACTOME_INTEGRATION_OF_ENERGY_METABOLISM
REACTOME_METABOLISM_OF_MRNA
REACTOME_METABOLISM_OF_RNA
REACTOME_METABOLISM_OF_AMINO_ACIDS_AND_DERIVATIVES
CHEN_LIVER_METABOLISM_QTL_CIS
WONG_MITOCHONDRIA_GENE_MODULE
0.0
0.2
0.4
0.6
0.0
0.2
0.4
0.6
GeneRatio
p.adjust
0.8
0.9
Count
10
20
30

Table S4

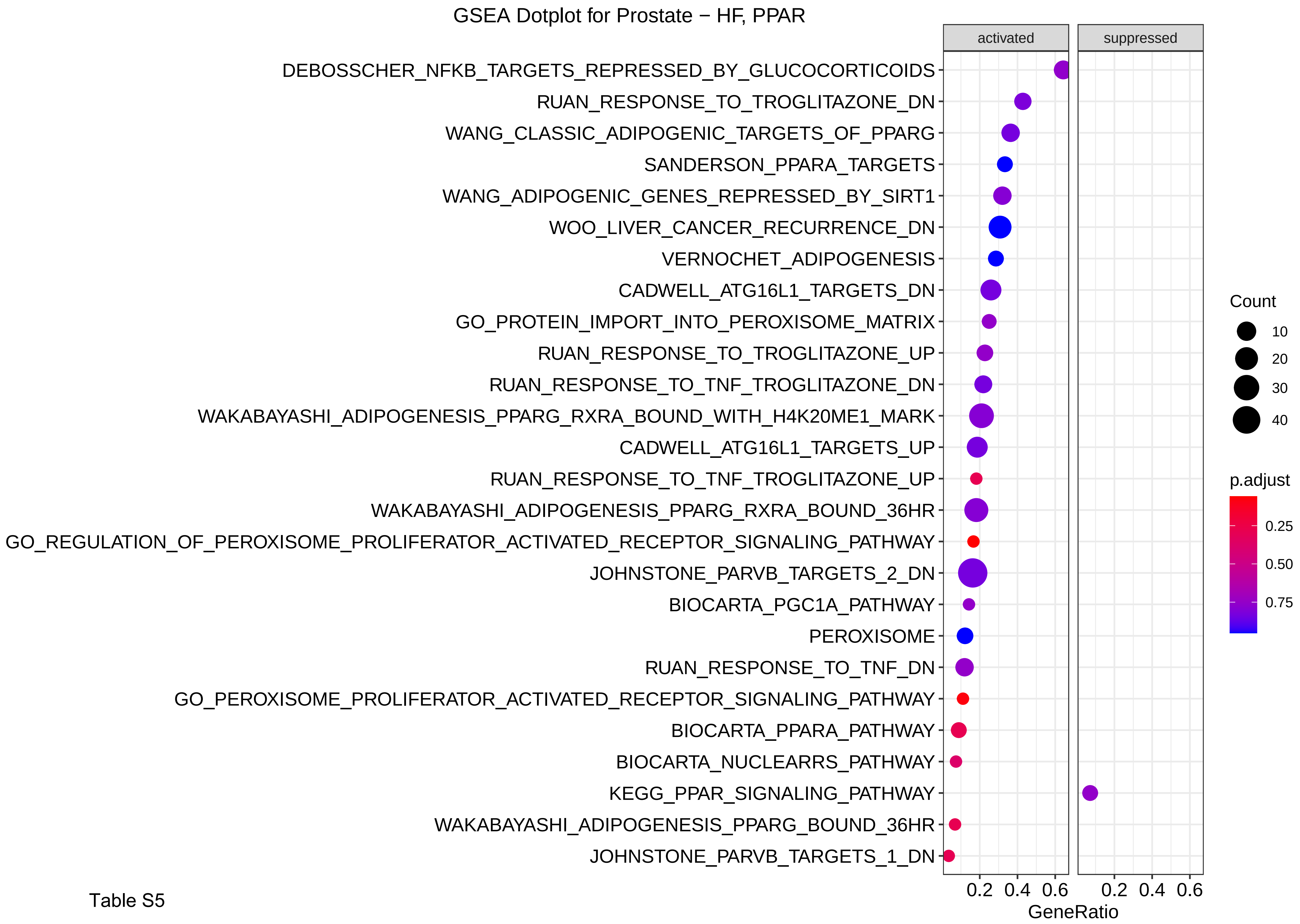

GSEA Dotplot for Prostate − HF, PPAR
activated
suppressed
DEBOSSCHER_NFKB_TARGETS_REPRESSED_BY_GLUCOCORTICOIDS
RUAN_RESPONSE_TO_TROGLITAZONE_DN
WANG_CLASSIC_ADIPOGENIC_TARGETS_OF_PPARG
SANDERSON_PPARA_TARGETS
WANG_ADIPOGENIC_GENES_REPRESSED_BY_SIRT1
WOO_LIVER_CANCER_RECURRENCE_DN
VERNOCHET_ADIPOGENESIS
CADWELL_ATG16L1_TARGETS_DN
GO_PROTEIN_IMPORT_INTO_PEROXISOME_MATRIX
RUAN_RESPONSE_TO_TROGLITAZONE_UP
RUAN_RESPONSE_TO_TNF_TROGLITAZONE_DN
WAKABAYASHI_ADIPOGENESIS_PPARG_RXRA_BOUND_WITH_H4K20ME1_MARK
CADWELL_ATG16L1_TARGETS_UP
RUAN_RESPONSE_TO_TNF_TROGLITAZONE_UP
WAKABAYASHI_ADIPOGENESIS_PPARG_RXRA_BOUND_36HR
GO_REGULATION_OF_PEROXISOME_PROLIFERATOR_ACTIVATED_RECEPTOR_SIGNALING_PATHWAY
JOHNSTONE_PARVB_TARGETS_2_DN
BIOCARTA_PGC1A_PATHWAY
PEROXISOME
RUAN_RESPONSE_TO_TNF_DN
GO_PEROXISOME_PROLIFERATOR_ACTIVATED_RECEPTOR_SIGNALING_PATHWAY
BIOCARTA_PPARA_PATHWAY
BIOCARTA_NUCLEARRS_PATHWAY
KEGG_PPAR_SIGNALING_PATHWAY
WAKABAYASHI_ADIPOGENESIS_PPARG_BOUND_36HR
JOHNSTONE_PARVB_TARGETS_1_DN
0.2 0.4 0.6
0.2 0.4 0.6
GeneRatio
Count
10
20
30
40
p.adjust
0.25
0.50
0.75

Table S5

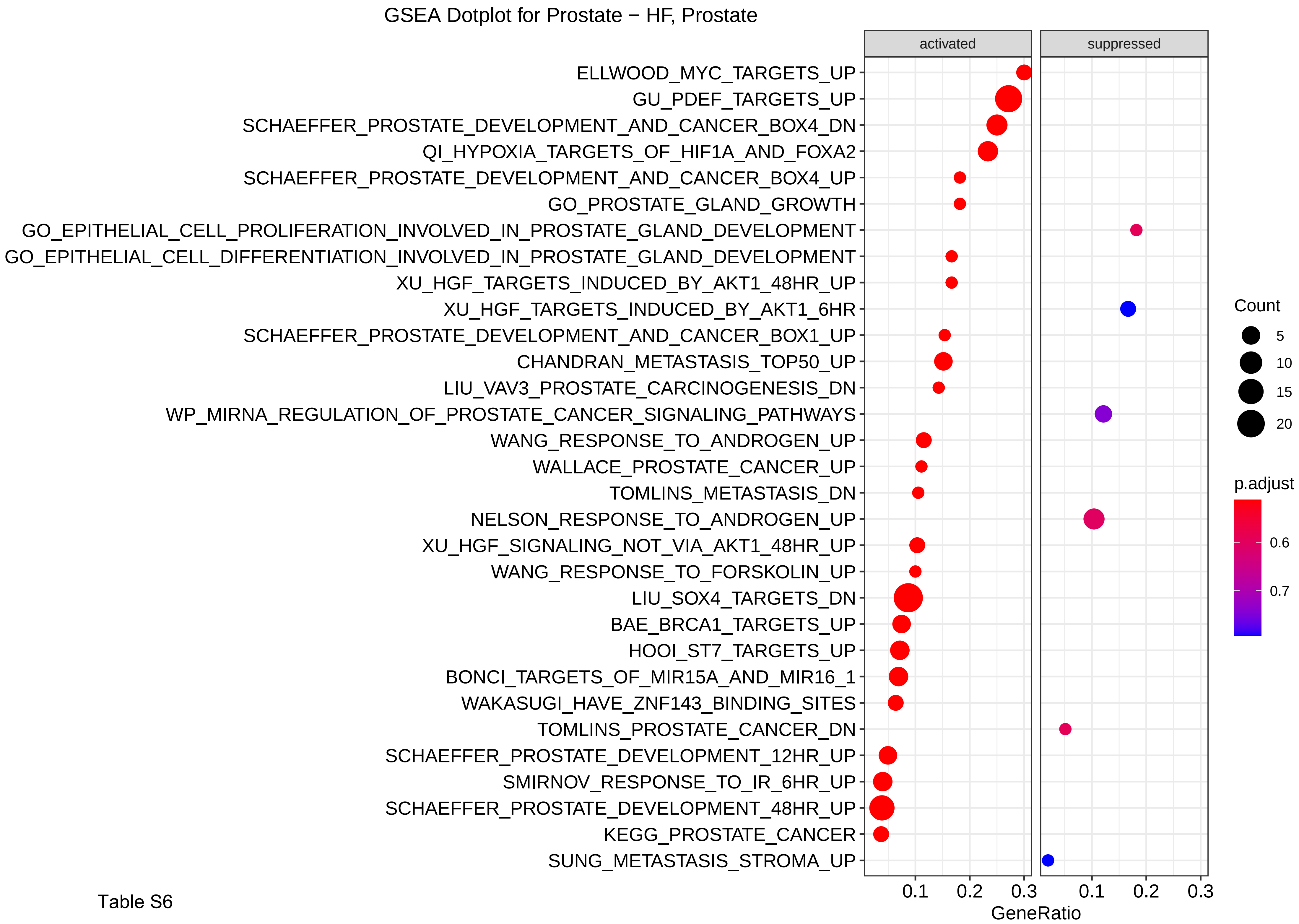

GSEA Dotplot for Prostate − HF, Prostate
activated
suppressed
ELLWOOD_MYC_TARGETS_UP
GU_PDEF_TARGETS_UP
SCHAEFFER_PROSTATE_DEVELOPMENT_AND_CANCER_BOX4_DN
QI_HYPOXIA_TARGETS_OF_HIF1A_AND_FOXA2
SCHAEFFER_PROSTATE_DEVELOPMENT_AND_CANCER_BOX4_UP
GO_PROSTATE_GLAND_GROWTH
GO_EPITHELIAL_CELL_PROLIFERATION_INVOLVED_IN_PROSTATE_GLAND_DEVELOPMENT
GO_EPITHELIAL_CELL_DIFFERENTIATION_INVOLVED_IN_PROSTATE_GLAND_DEVELOPMENT
XU_HGF_TARGETS_INDUCED_BY_AKT1_48HR_UP
XU_HGF_TARGETS_INDUCED_BY_AKT1_6HR
SCHAEFFER_PROSTATE_DEVELOPMENT_AND_CANCER_BOX1_UP
CHANDRAN_METASTASIS_TOP50_UP
LIU_VAV3_PROSTATE_CARCINOGENESIS_DN
WP_MIRNA_REGULATION_OF_PROSTATE_CANCER_SIGNALING_PATHWAYS
WANG_RESPONSE_TO_ANDROGEN_UP
WALLACE_PROSTATE_CANCER_UP
TOMLINS_METASTASIS_DN
NELSON_RESPONSE_TO_ANDROGEN_UP
XU_HGF_SIGNALING_NOT_VIA_AKT1_48HR_UP
WANG_RESPONSE_TO_FORSKOLIN_UP
LIU_SOX4_TARGETS_DN
BAE_BRCA1_TARGETS_UP
HOOI_ST7_TARGETS_UP
BONCI_TARGETS_OF_MIR15A_AND_MIR16_1
WAKASUGI_HAVE_ZNF143_BINDING_SITES
TOMLINS_PROSTATE_CANCER_DN
SCHAEFFER_PROSTATE_DEVELOPMENT_12HR_UP
SMIRNOV_RESPONSE_TO_IR_6HR_UP
SCHAEFFER_PROSTATE_DEVELOPMENT_48HR_UP
KEGG_PROSTATE_CANCER
SUNG_METASTASIS_STROMA_UP
0.1
0.2
0.3
0.1
0.2
0.3
GeneRatio
Count
5
10
15
20
p.adjust
0.6
0.7

Table S6

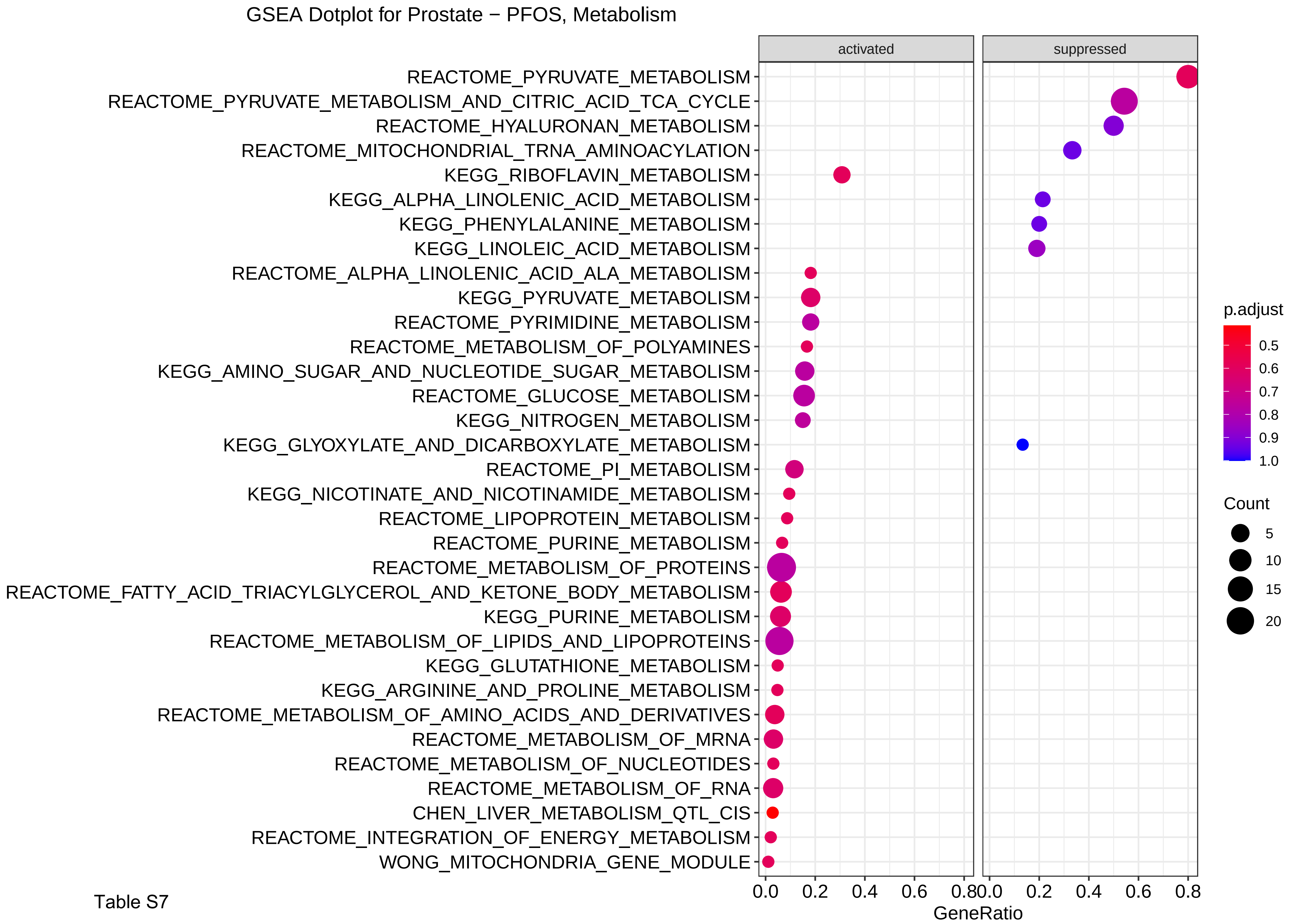

GSEA Dotplot for Prostate − PFOS, Metabolism
activated
suppressed
REACTOME_PYRUVATE_METABOLISM
REACTOME_PYRUVATE_METABOLISM_AND_CITRIC_ACID_TCA_CYCLE
REACTOME_HYALURONAN_METABOLISM
REACTOME_MITOCHONDRIAL_TRNA_AMINOACYLATION
KEGG_RIBOFLAVIN_METABOLISM
KEGG_ALPHA_LINOLENIC_ACID_METABOLISM
KEGG_PHENYLALANINE_METABOLISM
KEGG_LINOLEIC_ACID_METABOLISM
REACTOME_ALPHA_LINOLENIC_ACID_ALA_METABOLISM
KEGG_PYRUVATE_METABOLISM
REACTOME_PYRIMIDINE_METABOLISM
REACTOME_METABOLISM_OF_POLYAMINES
KEGG_AMINO_SUGAR_AND_NUCLEOTIDE_SUGAR_METABOLISM
REACTOME_GLUCOSE_METABOLISM
KEGG_NITROGEN_METABOLISM
KEGG_GLYOXYLATE_AND_DICARBOXYLATE_METABOLISM
REACTOME_PI_METABOLISM
KEGG_NICOTINATE_AND_NICOTINAMIDE_METABOLISM
REACTOME_LIPOPROTEIN_METABOLISM
REACTOME_PURINE_METABOLISM
REACTOME_METABOLISM_OF_PROTEINS
REACTOME_FATTY_ACID_TRIACYLGLYCEROL_AND_KETONE_BODY_METABOLISM
KEGG_PURINE_METABOLISM
REACTOME_METABOLISM_OF_LIPIDS_AND_LIPOPROTEINS
KEGG_GLUTATHIONE_METABOLISM
KEGG_ARGININE_AND_PROLINE_METABOLISM
REACTOME_METABOLISM_OF_AMINO_ACIDS_AND_DERIVATIVES
REACTOME_METABOLISM_OF_MRNA
REACTOME_METABOLISM_OF_NUCLEOTIDES
REACTOME_METABOLISM_OF_RNA
CHEN_LIVER_METABOLISM_QTL_CIS
REACTOME_INTEGRATION_OF_ENERGY_METABOLISM
WONG_MITOCHONDRIA_GENE_MODULE
0.0 0.2 0.4 0.6 0.8
0.0 0.2 0.4 0.6 0.8
GeneRatio
p.adjust
0.5
0.6
0.7
0.8
0.9
1.0
Count
5
10
15
20

Table S7

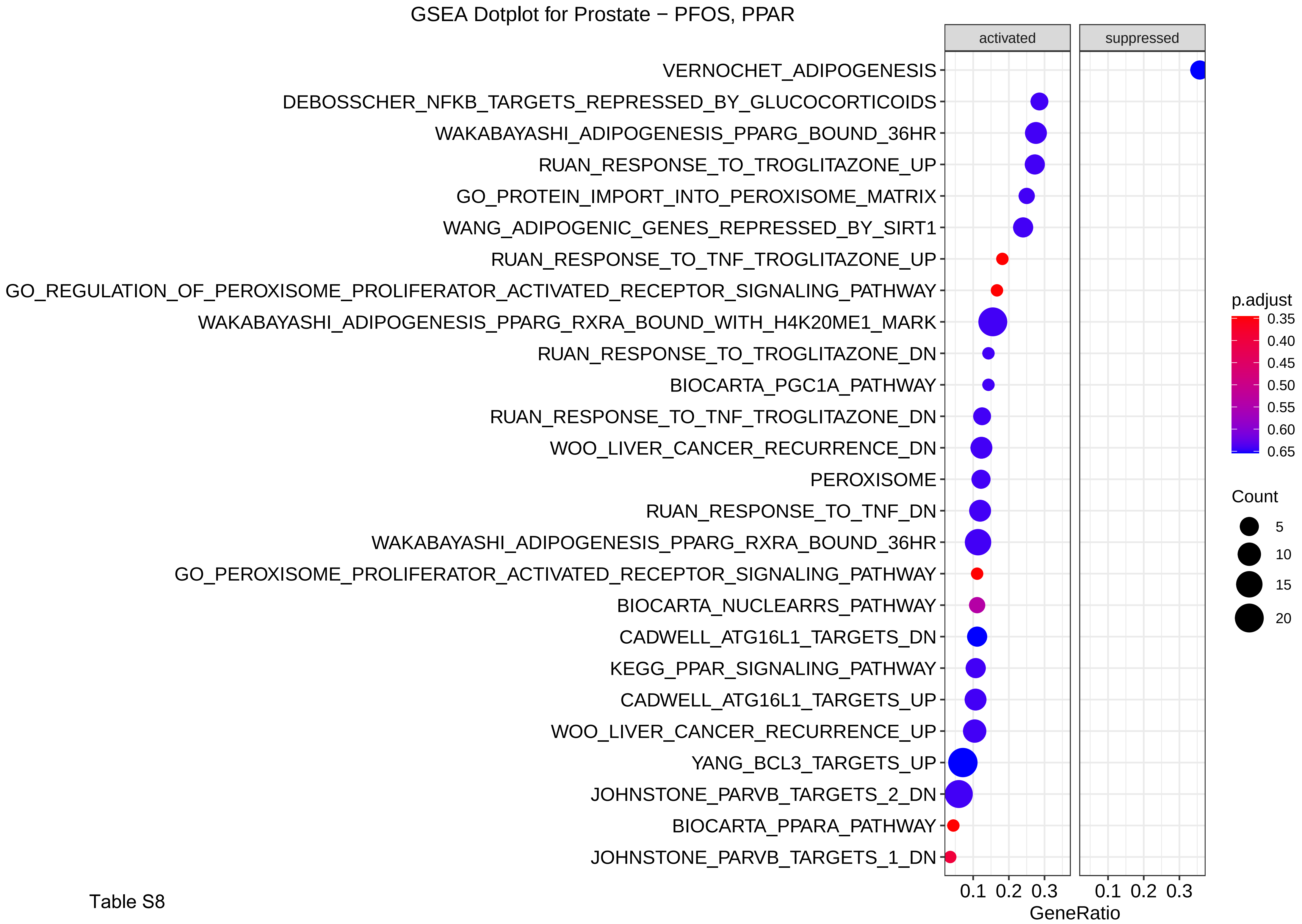
GSEA Dotplot for Prostate − PFOS, PPAR
activated
suppressed
VERNOCHET_ADIPOGENESIS
DEBOSSCHER_NFKB_TARGETS_REPRESSED_BY_GLUCOCORTICOIDS
WAKABAYASHI_ADIPOGENESIS_PPARG_BOUND_36HR
RUAN_RESPONSE_TO_TROGLITAZONE_UP
GO_PROTEIN_IMPORT_INTO_PEROXISOME_MATRIX
WANG_ADIPOGENIC_GENES_REPRESSED_BY_SIRT1
RUAN_RESPONSE_TO_TNF_TROGLITAZONE_UP
GO_REGULATION_OF_PEROXISOME_PROLIFERATOR_ACTIVATED_RECEPTOR_SIGNALING_PATHWAY
WAKABAYASHI_ADIPOGENESIS_PPARG_RXRA_BOUND_WITH_H4K20ME1_MARK
RUAN_RESPONSE_TO_TROGLITAZONE_DN
BIOCARTA_PGC1A_PATHWAY
RUAN_RESPONSE_TO_TNF_TROGLITAZONE_DN
WOO_LIVER_CANCER_RECURRENCE_DN
PEROXISOME
RUAN_RESPONSE_TO_TNF_DN
WAKABAYASHI_ADIPOGENESIS_PPARG_RXRA_BOUND_36HR
GO_PEROXISOME_PROLIFERATOR_ACTIVATED_RECEPTOR_SIGNALING_PATHWAY
BIOCARTA_NUCLEARRS_PATHWAY
CADWELL_ATG16L1_TARGETS_DN
KEGG_PPAR_SIGNALING_PATHWAY
CADWELL_ATG16L1_TARGETS_UP
WOO_LIVER_CANCER_RECURRENCE_UP
YANG_BCL3_TARGETS_UP
JOHNSTONE_PARVB_TARGETS_2_DN
BIOCARTA_PPARA_PATHWAY
JOHNSTONE_PARVB_TARGETS_1_DN
0.1 0.2 0.3
0.1 0.2 0.3
GeneRatio
p.adjust
0.35
0.40
0.45
0.50
0.55
0.60
0.65
Count
5
10
15
20

Table S8

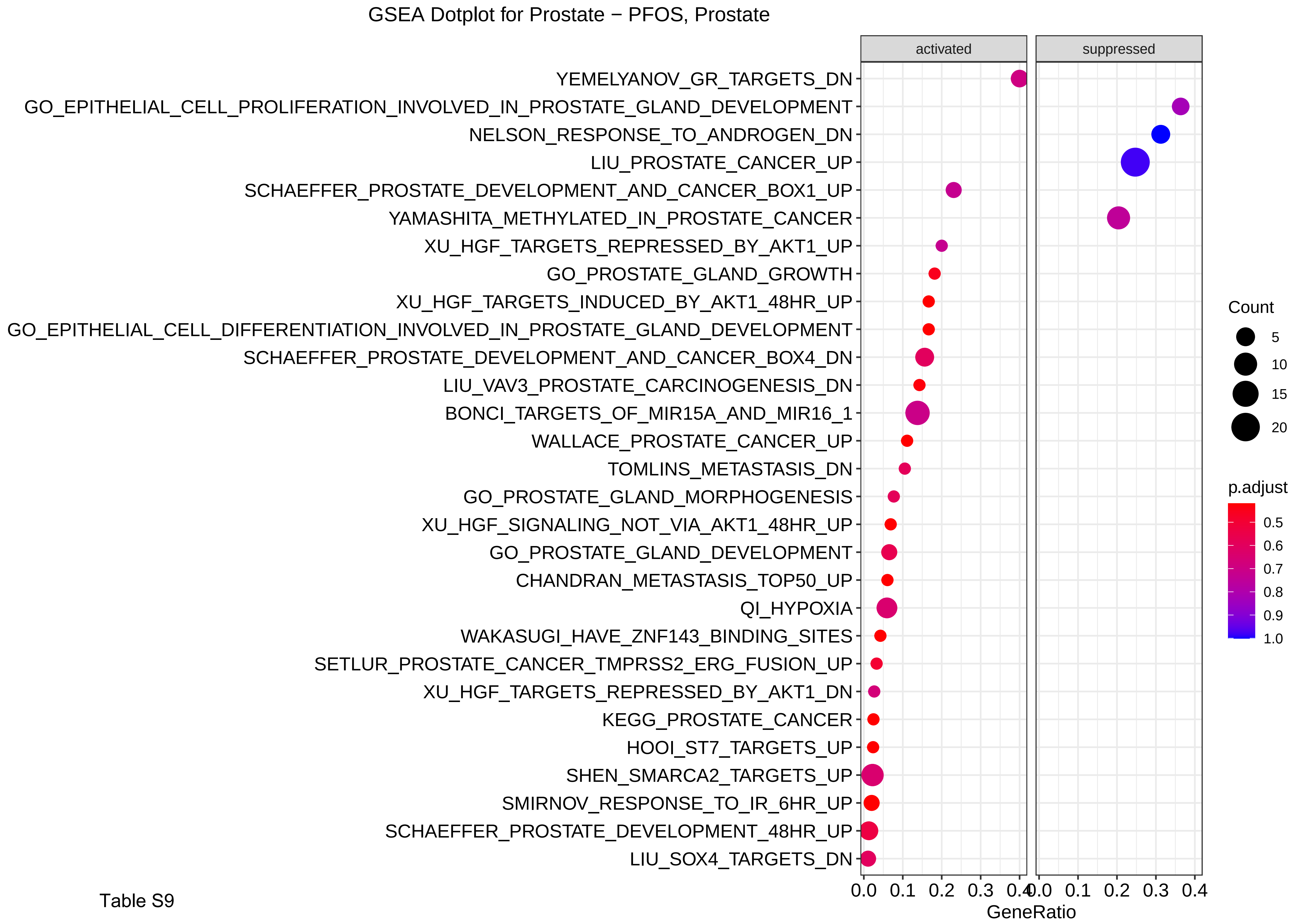
GSEA Dotplot for Prostate − PFOS, Prostate
activated
suppressed
YEMELYANOV_GR_TARGETS_DN
GO_EPITHELIAL_CELL_PROLIFERATION_INVOLVED_IN_PROSTATE_GLAND_DEVELOPMENT
NELSON_RESPONSE_TO_ANDROGEN_DN
LIU_PROSTATE_CANCER_UP
SCHAEFFER_PROSTATE_DEVELOPMENT_AND_CANCER_BOX1_UP
YAMASHITA_METHYLATED_IN_PROSTATE_CANCER
XU_HGF_TARGETS_REPRESSED_BY_AKT1_UP
GO_PROSTATE_GLAND_GROWTH
XU_HGF_TARGETS_INDUCED_BY_AKT1_48HR_UP
GO_EPITHELIAL_CELL_DIFFERENTIATION_INVOLVED_IN_PROSTATE_GLAND_DEVELOPMENT
SCHAEFFER_PROSTATE_DEVELOPMENT_AND_CANCER_BOX4_DN
LIU_VAV3_PROSTATE_CARCINOGENESIS_DN
BONCI_TARGETS_OF_MIR15A_AND_MIR16_1
WALLACE_PROSTATE_CANCER_UP
TOMLINS_METASTASIS_DN
GO_PROSTATE_GLAND_MORPHOGENESIS
XU_HGF_SIGNALING_NOT_VIA_AKT1_48HR_UP
GO_PROSTATE_GLAND_DEVELOPMENT
CHANDRAN_METASTASIS_TOP50_UP
QI_HYPOXIA
WAKASUGI_HAVE_ZNF143_BINDING_SITES
SETLUR_PROSTATE_CANCER_TMPRSS2_ERG_FUSION_UP
XU_HGF_TARGETS_REPRESSED_BY_AKT1_DN
KEGG_PROSTATE_CANCER
HOOI_ST7_TARGETS_UP
SHEN_SMARCA2_TARGETS_UP
SMIRNOV_RESPONSE_TO_IR_6HR_UP
SCHAEFFER_PROSTATE_DEVELOPMENT_48HR_UP
LIU_SOX4_TARGETS_DN
0.0 0.1 0.2 0.3 0.4
0.0 0.1 0.2 0.3 0.4
GeneRatio
Count
5
10
15
20
p.adjust
0.5
0.6
0.7
0.8
0.9
1.0

Table S9
